# Editorial: Bioinformatics of genome regulation and systems biology, Volume III

**DOI:** 10.3389/fgene.2023.1215987

**Published:** 2023-05-18

**Authors:** Anastasia A. Anashkina, Nina G. Orlova, Alexander N. Ignatov, Ming Chen, Yuriy L. Orlov

**Affiliations:** ^1^ The Digital Health Institute, I.M.Sechenov First Moscow State Medical University of the Russian Ministry of Health (Sechenov University), Moscow, Russia; ^2^ Engelhardt Institute of Molecular Biology, Russian Academy of Sciences, Moscow, Russia; ^3^ Department of Mathematics, Financial University Under the Government of the Russian Federation, Moscow, Russia; ^4^ Agrarian and Technological Institute, Peoples’ Friendship University of Russia (RUDN University), Moscow, Russia; ^5^ Department of Bioinformatics, College of Life Sciences, Zhejiang University, Hangzhou, China; ^6^ Institute of Cytology and Genetics, Siberian Branch of the Russian Academy of Sciences, Novosibirsk, Russia; ^7^ Institute of Life Sciences and Biomedicine, Far East Federal University, Vladivostok, Russia

**Keywords:** bioinformatics, gene expression, regulation, systems biology, cancer biology, Editorial

This Research Topic, “*Bioinformatics of genome regulation and systems biology, Volume III*,” continues the studies in the field of bioinformatics of gene expression presented initially in *Frontiers in Genetics* journal (https://www.frontiersin.org/research-topics/8383/bioinformatics-of-genome-regulation-and-systems-biology) and then in the Volumes I and II (Orlov et al., 2021a; Orlov et al., 2021c) (https://www.frontiersin.org/research-topics/14266/bioinformatics-of-genome-regulation-volume-i). The materials presented in these Research Topics were initially discussed in a BGRS\SB (Bioinformatics of Genome Regulation and Structure\Systems Biology) conference series in Novosibirsk, Russia (https://bgrssb.icgbio.ru/2022/) (Kolchanov and Orlov, 2013; Orlov et al., 2015), and then complemented by new works on bioinformatics and computational genomics. Gene expression regulation at the genome level remains the focus of the published papers. Materials of the conferences on genetics and genomics were presented in *Frontiers in Genetics* (Orlov and Baranova, 2020). Due to popular demand, the Research Topics were extended as Volume II (Orlov et al., 2021a; Orlov et al., 2021b) and then in this Volume III. The BGRS conference series materials on bioinformatics and gene expression regulation at gene, chromosome, and genome levels have been widely presented in the special journal Research Topic (Orlov et al., 2016; Orlov et al., 2019; Tatarinova et al., 2019). Recently, *Frontiers in Genetics* Research Topics continue discussion of these science problems (Tatarinova et al., 2019; Orlov et al., 2021b; Das et al., 2022; Orlov et al., 2022). We have to acknowledge continuing the thematic journal Research Topic “Bioinformatics of Gene Regulations” (Orlov et al., 2021b) at MDPI IJMS (https://www.mdpi.com/journal/ijms/special_issues/MVA479KFR7).

This Research Topic presents eight papers on medical genomics and new bioinformatics tool methods. Dvorak et al. discussed the effect of intron position on gene expression regulation in their Brief research report article. Longer first introns were found to be a general property of eukaryotic gene structure and shown to contain a higher fraction of conserved sequence and different functional elements. The authors argued that the position of the longest intron in a gene can regulate gene expression.

Deyneko in his Opinion article discussed the evaluation of motif recognition methods in bioinformatics. The author suggested the performance metrics to test motif analysis software. Another methodical work was presented by Li et al. The qPCRtools package enables users to analyze the efficiency of gene amplification in qPCR data.

Biomedical papers present applications of bioinformatics modeling to different cancer studies. Chen et al. studied the prognostic regulatory role of necroptosis in clear cell renal cell carcinoma. The necroptosis-related subtypes were identified by mining the public cohort obtained from The Cancer Genome Atlas. The clinical implication of the developed score was further validated in the patients’ cohort. Luan et al. investigated the usefulness of 5-methylcytosine (5 mC) in hepatocellular carcinoma. An unsupervised clustering method was used to identify novel subtypes of hepatocellular carcinoma based on the expression of 5-methylcytosine gene signatures. The 5 mC score developed was effective in the clinical response prediction to several treatment options, including targeted therapy. Through a machine learning approach, the authors identified irinotecan (topoisomerase I inhibitor) (Xu et al., 2017) as a potentially promising drug for the treatment of hepatocellular carcinoma patients. Gu et al. studied hub genes and pathways associated with mitochondrial dysfunction in hypertrophy of ligamentum flavum.

Finally, a group of papers discovered the role of miRNA in cancer. Akimniyazova et al. studied the regulation of gene expression in esophageal adenocarcinoma by piRNAs (PIWI-interacting RNA). The authors considered the role of piRNAs as efficient regulators of mRNA translation (Cai et al., 2022) for esophageal adenocarcinoma candidate genes using the bioinformatics methods for piRNA–mRNA interaction prediction. The work is an extension of the previously published Research Topic on Genome Expression Regulation by the same scientific team (Mukushkina et al., 2020). Nersisyan et al. considered a novel approach for a joint analysis of isomiR and mRNA expression data in breast cancer. The application of small RNA sequencing technology led researchers to identify miRNA isoforms (isomiRs): variants of a mature miRNA differing from each other by a few nucleotides. The authors observed strong cancer subtype-specific patterns of isomiR activity, highlighting the differences between breast cancer molecular subtypes and normal tissues. This work continues the studies on miRNA regulatory interactions in cancer presented previously by the same group (Shkurnikov et al., 2019).

Overall, we are proud of the continuing Research Topics at *Frontiers in Genetics* we collated. Biomedical applications focusing on gene expression in chronic diseases are discussed in a new Research Topic, “High-throughput sequencing-based investigation of chronic disease markers and mechanisms - volume II” (https://www.frontiersin.org/research-topics/53085/high-throughput-sequencing-based-investigation-of-chronic-disease-markers-and-mechanisms---volume-ii) (Orlov et al., 2022). Computational plant biology applications will be considered in “Applications of Artificial Intelligence, Machine Learning, and Deep Learning in Plant Breeding” Research Topic (https://www.frontiersin.org/research-topics/54136/applications-of-artificial-intelligence-machine-learning-and-deep-learning-in-plant-breeding). We hope you will find this Research Topic useful to overview frontier studies in computational genomics.

## References

[B1] Cai A. Hu Y. Zhou Z. Qi Q. Wu Y. Dong P. (2022). PIWI-interacting RNAs (piRNAs): Promising applications as emerging biomarkers for digestive system cancer. Front. Mol. Biosci. 27 (9), 848105. 10.3389/fmolb.2022.848105 PMC8829394 35155584

[B2] Das R. Tatarinova T. V. Galieva E. R. Orlov Y. L. (2022). Editorial: Association between individuals’ genomic ancestry and variation in disease susceptibility. Front. Genet. 13, 831320. 10.3389/fgene.2022.831320 35186044 PMC8847436

[B3] Kolchanov N. A. Orlov Y. L. (2013). Introductory note for BGRS-2012 special issue. J. Bioinforma. Comput. Biol. 11 (1), 1302001. 10.1142/S0219720013020010 28847203

[B4] Mukushkina D. Aisina D. Pyrkova A. Ryskulova A. Labeit S. Ivashchenko A. (2020). *In silico* prediction of miRNA interactions with candidate atherosclerosis gene mRNAs. Front. Genet. 11, 605054. 10.3389/fgene.2020.605054 33329752 PMC7672156

[B5] Orlov Y. L. Anashkina A. A. Klimontov V. V. Baranova A. V. (2021a). Medical genetics, genomics and bioinformatics aid in understanding molecular mechanisms of human diseases. Int. J. Mol. Sci. 22, 9962. 10.3390/ijms22189962 34576125 PMC8467458

[B6] Orlov Y. L. Anashkina A. A. Tatarinova T. V. Baranova A. V. (2021b). Editorial: Bioinformatics of genome regulation, volume II. Front. Genet. 12, 795257. 10.3389/fgene.2021.795257 34819949 PMC8606529

[B7] Orlov Y. L. Baranova A. V. (2020). Editorial: Bioinformatics of genome regulation and Systems biology. Front. Genet. 11, 625. 10.3389/fgene.2020.00625 32849761 PMC7399369

[B8] Orlov Y. L. Baranova A. V. Markel A. L. (2016). Computational models in genetics at BGRS\SB-2016: Introductory note. BMC Genet. 17 (3), 155. 10.1186/s12863-016-0465-3 28105935 PMC5249000

[B9] Orlov Y. L. Chen W. L. Sekacheva M. I. Cai G. Li H. (2022). Editorial: High-Throughput sequencing-based investigation of chronic disease markers and mechanisms. Front. Genet. 13, 922206. 10.3389/fgene.2022.922206 35801080 PMC9253685

[B10] Orlov Y. L. Hofestädt R. M. Kolchanov N. A. (2015). Introductory note for BGRS\SB-2014 special issue. J. Bioinform. Comput. Biol. 13, 1502001. 10.1142/S0219720015020011 25666651

[B11] Orlov Y. L. Hofestädt R. Tatarinova T. V. (2019). Bioinformatics research at BGRS \ SB-2018. J. Bioinform. Comput. Biol. 17, 1902001. 10.1142/S0219720019020013 30866731

[B12] Orlov Y. L. Tatarinova T. V. Oparina N. Y. Galieva E. R. Baranova A. V. (2021c). Editorial: Bioinformatics of genome regulation, volume I. Front. Genet. 12, 803273. 10.3389/fgene.2021.803273 34938326 PMC8687738

[B15] Shkurnikov M. Nikulin S. Nersisyan S. Poloznikov A. Zaidi S. Baranova A. (2019). LAMA4-regulating miR-4274 and its host gene SORCS2 play a role in IGFBP6-dependent effects on phenotype of basal-like breast cancer. Front. Mol. Biosci. 6, 122. 10.3389/fmolb.2019.00122 31781574 PMC6857517

[B13] Tatarinova T. V. Chen M. Orlov Y. L. (2019). Bioinformatics research at BGRS-2018. BMC Bioinforma. 20 (1), 33. 10.1186/s12859-018-2566-7 PMC6362573 30717672

[B14] Xu L. Zhu Y. Shao J. Chen M. Yan H. Li G. (2017). Dasatinib synergises with irinotecan to suppress hepatocellular carcinoma via inhibiting the protein synthesis of PLK1. Br. J. Cancer 116 (8), 1027–1036. 10.1038/bjc.2017.55 28267710 PMC5396112

